# Semi-supervised COVID-19 CT image segmentation using deep generative models

**DOI:** 10.1186/s12859-022-04878-6

**Published:** 2022-08-17

**Authors:** Judah Zammit, Daryl L. X. Fung, Qian Liu, Carson Kai-Sang Leung, Pingzhao Hu

**Affiliations:** 1 Department of Computer Science, University of Manitoba, Winnipeg, MB R3T 2N2 Canada; 2 Department of Biochemistry and Medical Genetics, University of Manitoba, Room 308 - Basic Medical Sciences Building, 745 Bannatyne Avenue, Winnipeg, MB R3E 0J3 Canada; 3 CancerCare Manitoba Research Institute, Winnipeg, MB Canada

**Keywords:** Semi-supervised learning, Convolutional network, Image segmentation, COVID-19, Computed tomography

## Abstract

**Background:**

A recurring problem in image segmentation is a lack of labelled data. This problem is especially acute in the segmentation of lung computed tomography (CT) of patients with Coronavirus Disease 2019 (COVID-19). The reason for this is simple: the disease has not been prevalent long enough to generate a great number of labels. Semi-supervised learning promises a way to learn from data that is unlabelled and has seen tremendous advancements in recent years. However, due to the complexity of its label space, those advancements cannot be applied to image segmentation. That being said, it is this same complexity that makes it extremely expensive to obtain pixel-level labels, making semi-supervised learning all the more appealing. This study seeks to bridge this gap by proposing a novel model that utilizes the image segmentation abilities of deep convolution networks and the semi-supervised learning abilities of generative models for chest CT images of patients with the COVID-19.

**Results:**

We propose a novel generative model called the shared variational autoencoder (SVAE). The SVAE utilizes a five-layer deep hierarchy of latent variables and deep convolutional mappings between them, resulting in a generative model that is well suited for lung CT images. Then, we add a novel component to the final layer of the SVAE which forces the model to reconstruct the input image using a segmentation that must match the ground truth segmentation whenever it is present. We name this final model StitchNet.

**Conclusion:**

We compare StitchNet to other image segmentation models on a high-quality dataset of CT images from COVID-19 patients. We show that our model has comparable performance to the other segmentation models. We also explore the potential limitations and advantages in our proposed algorithm and propose some potential future research directions for this challenging issue.

## Background

Modern deep learning based image segmentation techniques tend to require vast amounts of pixel-level labels to be effective. However, to obtain these labels, it is necessary to have someone sit down and categorize every pixel in an image. This requires a massive amount of human effort. In the case of biomedical images, this is made worse by the fact that it is often necessary to have a panel of experts do the labelling. Therefore, any technique that has the potential to reduce the number of labelled images needed has immense value.

This issue can be seen in the diagnosis and prognosis of patients suspected to have the Coronavirus Disease 2019 (COVID-19), using computed tomography (CT) scans of their lungs. A pixel-wise segmentation of these scans, identifying healthy tissue as well as parts of the lungs affected by either common pneumonia or novel coronavirus pneumonia, can be a powerful tool for diagnosis as well as for identifying how much risk the patient is in, or will be in. Obtaining these segmentations, however, is immensely time-consuming for medical professionals to do by hand. In response to this, there has been work [1–3] in using deep learning models for image segmentation to automate this process. Despite there being massive datasets of CT images, these models can only be trained on CT images that have been hand labelled by skilled radiologists, severely limiting the amount of usable data.

Semi-supervised learning has the potential to alleviate this issue. A semi-supervised model has the ability to learn from both unlabelled and labelled images simultaneously, drastically reducing the number of labelled images needed to achieve satisfactory performance. For this reason, there has been a surge of research into semi-supervised learning in recent years. We will discuss some notable previous work in image segmentation, starting with several fully-supervised models and following with several semi-supervised models.

U-Net [4] is a deep learning based image segmentation model that has seen great success on medical imagery tasks. It utilizes an encoder-decoder style architecture with skip connections between the encoder and the decoder. SegNet [5] has a similar encoder-decoder style architecture, however instead of skip connections it uses max unpooling layers in the decoder. The MobileNetV2 [6], an image classification network, can be used as the U-Net’s encoder. When compared to much larger encoder networks, the MobileNetV2 achieves only slightly worse performance while being much faster. Zhang et al. [1] have used several deep learning based, supervised segmentation models [4, 5, 7] to predict a segmentation for a CT image of a patient’s lungs. Fan et al. [3] and Chen et al. [8] both propose novel supervised segmentation models that have been handcrafted to perform well on chest CT images. Though impressive, these models are still limited by the number of CT images with pixel-level labels.

Moving away from fully supervised models, there is a plethora of papers proposing deep learning models that use image-level labels as a supervisory signal for the task of image segmentation. They *do not* utilize completely unlabelled images. This task is sometimes referred to as pure or true semi-supervision, and there are precious few published papers that tackle it [9–19].

The above purely semi-supervised models tend to tackle the problem using some form of adversarial training, self-training, clustering or multi-view training. Many papers [10, 13, 16, 18, 19] use an adversarially trained discriminator deep convolutional network to ensure that the prediction of some segmentation model is realistic. This scheme allows them to train their network on unlabelled photos by leveraging the fact that, even if you do not know the ground truth, it should at least belong to the same distribution as the ground truth for the labelled images. The main drawback to this technique is that it can be very difficult to get a model with an adversarial component to converge to a solution.

Other papers [9, 14] use the fact that images–labelled or unlabelled–that have been determined to be similar by some deep learning-based, unsupervised clustering algorithm should also be close to each other in various latent and feature spaces. These techniques are dependent on how you define “close” which can be quite difficult for data that is as high dimensional as images, causing the performance of these models to be underwhelming.

Pseudo-labelling [20] is a commonly used semi-supervised learning technique where a fully supervised deep network is trained and then used to make predictions on some unlabelled data. The network is then retrained using the model’s most confident predictions as labels. However, if this prediction is of low quality, then this scheme will continuously reinforce this bad behaviour to disastrous effect. As a result, pseudo-labelling is typically considered the least effective, but simplest, semi-supervised technique. There are several papers [3, 12] that use this general scheme with some significant modifications.

As with this paper, many papers [2, 3] seek to utilize unlabelled CT images from COVID-19 patients. Shan et al. [2] use an intriguing human-in-the-loop strategy. This strategy entails training a deep learning-based, segmentation network on a small dataset of pixel-wise labelled data, then using this network to make prediction on a large unlabelled dataset. These predictions are then refined by a skilled radiologist and included in the pixel-level labelled dataset. The network is retrained, and this process repeats until satisfactory performance is achieved. Though the labelling effort is significantly reduced, this technique still requires some manual labelling effort and many research groups will simply *not* have access to a radiologist.

Fan et al. [3] use pseudo-labelling in its most rudimentary form. Despite this, they achieved a sizable increase in segmentation performance compared to their fully supervised baseline. This makes it quite motivating to employ a more sophisticated semi-supervised technique, as pseudo-labelling is far from capable of making full use of these unlabelled images. Fung et al. [21] proposed a model that does just this. They add a self-supervised pre-training step to Fan et al.’s InfNet model. During this step, the CT images are obscured with a black rectangle and the model is trained to reconstruct the full CT image. Though this method was able to improve on the InfNet, it is trained in two separate steps and a semi-supervised technique that can be trained end-to-end may improve the performance even further.

Deep generative models offer an elegant framework for semi-supervised models. In essence, they treat the image and label as two random variables in a graphical model and seek to model both using recent advances in variational inference. Some notable examples are the M2 variational autoencoder [22] and the auxiliary deep generative model [23]. Unfortunately, the vast majority of this research has been in the domain of image classification, where the label is simply a single category for each image. The assumption can be made that each of these categories are equally likely to occur. Even though this assumption is very close to the reality, it still allows for easy to compute, closed-form calculations. Modern deep generative-based, semi-supervised techniques rely heavily on this fact.

A similar assumption *cannot* be made in the case of image segmentation. This is for several critical reasons. First, due to the fact that *each* pixel has a label, the number of unique segmentations is exponentially larger than the number of unique image-level labels. Furthermore, very few of these unique segmentations are realistic. For example, a set of pixels that have been give the *dog* label but are in the shape of a human is not a realistic segmentation. This is important because it completely removes our ability to assume that each unique segmentation is equally likely to occur. Finally, the label for each pixel is heavily dependent on the labels of the other pixels in the image, removing the possibility of making any independence assumptions. For these reasons, modern semi-supervised techniques tend to fall flat when used for image segmentation.

Though problematic, the issues mentioned above are not at all new. The same issues are encountered while trying to find a distribution capable of modelling images. The variational approach [24, 25] handles this by finding a latent representation of the image as well as a deep learning-based, functional mapping between the image and its latent representation. You are then free to make simplifying assumptions about the latent space’s distribution without making any assumptions about the images’ distribution. In this study, we utilize this approach by finding a latent representation of both the original CT image, and its segmentation. Because we want our model to learn to segment images even when the ground-truth segmentation is not present, we assume that the original CT image is dependent on the segmentation. By doing this, in the absence of the ground-truth segmentation, the model learns to predict a segmentation that is useful for the reconstruction of the original CT image.

Though the original variational autoencoder (VAE) [24] could be used for this task, it lacks the expressivity to sufficiently model large datasets. This is particularly true when the dataset is one of images. The ladder variational autoencoder (LVAE) [26] greatly increases the expressivity of the VAE by introducing a hierarchy of latent variables and a novel way of training such a hierarchy. In this study, we modify the LVAE by sharing several key weights across the inference and generative network. Additionally, we replace all functional mappings in the LVAE with deep convolutional networks that have been handcrafted to work well on CT images. We name the resultant model the *shared variational autoencoder* (SVAE).

In their original forms, the VAE, LVAE and SVAE are designed to take an image as input and reconstruct that image as its output. We modify the SVAE to output both a segmentation mask and four CT images, one for each of the segmentation labels. Then, we reconstruct the original CT image by *stitching* together the four CT images based on the segmentation. We name the resultant model *StitchNet*. In summary, we develop a novel deep generative model called the SVAE. Then, using the SVAE, we create a semi-supervised model called StitchNet and test it on a high-quality dataset of CT images from COVID-19 patients.

## Results

### Dataset

For the evaluation of StitchNet, the Zhang et al.’s [1] China Consortium of Chest CT Image Investigation (CC-CCII), with several modifications, was used. CC-CCII contains CT images from 2,778 patients, totalling 444,034 images in total. Eighty-five percent of the patients were from the Chinese cities of Yichang, Hefei or Guangzhou and the remainder were from an international cohort. The patients either had common pneumonia (CP), novel-coronavirus pneumonia (NCP), or were part of the control group. In our dataset, we excluded the patients with CP.

CC-CCII contained 750 segmentation masks, which correspond to 150 patients with NCP. The segmentation was completed by five senior radiologists with over 25 years of experience. They segmented three labels: health lung field, ground-glass opacity and consolidation.

**Fig. 1:**
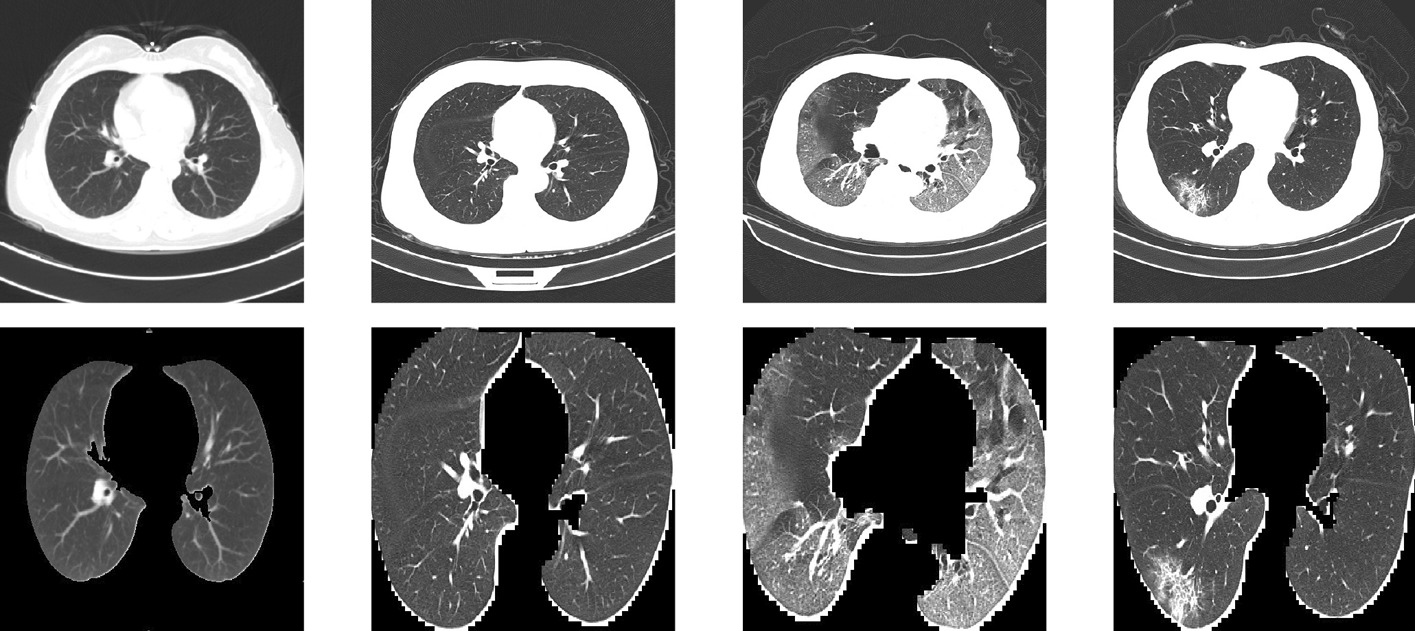
Before (top) and after (bottom) data pre-processing

We will now discuss the data pre-processing and cleaning procedure we employed. We segmented the lung field in each CT image using the U-Net semantic segmentation model. The opening and closing morphological transformations were used for noise reduction. The images were then cropped to only include lung field. The result is shown in Fig. 1. Before being used in our models, all images are resized to a resolution of 352⨯352 and the pixels values are scaled to be between zero and one. We randomly separate 60% of the labelled data into the training set, 20% into the testing set and 20% validation set. We do this by patient, not by image, so that all of a single patient’s CT images will be in exactly one of the three sets, thus avoiding data leakage.

### Evaluation metrics

For each image, we employ the following four evaluation metrics: the Intersection-over-Union, F1-Score, Recall and Precision.

(1) *The Intersection-over-Union (IoU)*: The Intersection-over-Union was used to measure the overlap between the ground-truth infected region (T) and the predicted infected region (P) in a way that controls for the size of the infected region.

(2) *The F1-Score (F1)*: The F1-Score, also called the Dice Coefficient, was used to measure the overlap between the ground-truth infected region (T) and the predicted infected region (P).

(3) *Recall (Rec.)*: The Recall, also called Sensitivity or True Positive Rate, was used to measure what proportion of the ground-truth infected region (T) was present in the predicted infected region (P).

(4) *Precision (Prec.)*: The Precision was used to measure what proportion of the predicted infected region (P) was present in the ground-truth infected region (T). These four metrics are defined in Eq. (1).

1 $$\begin{aligned} {\text {IoU}} = \frac{|T \cap P|}{|T \cup P|},\,\,\,\,\, {\text {F1}} = \frac{2 \cdot |T \cap P|}{|T| + |P|},\,\,\,\,\,{\text {Rec.}} = \frac{|T \cap P|}{|T|}, \,\,\,\,\, {\text {Prec.}} = \frac{|T \cap P|}{|P|}, \end{aligned}$$

where $|\,\,\,|$ is the operator that calculates the number of pixels in the given region, $\cap$ is the intersection operator, and $\cup$ is the union operator.

We calculate the above metrics for each CT image and average the results. The *Mean* and *Standard Deviation (STD)* are defined as follows:

Let *M*((*x*, *y*)) be the value of the relevant evaluation metric calculated for the data point (*x*, *y*). Then let ${\text {Metric}} = \{M((x_i,y_i))\} _{(x_i,y_i)}\in D_{Val}$, where $D_{Val}$ is the validation dataset.

(1) *Mean*: Then the *Mean* is simply $\underset{{\text {Metric}}}{\sum }\frac{M((x_i,y_i))}{|{\text {Metric}}|}$.

(2) *STD*: The STD is $\sqrt{\frac{1}{|{\text {Metric}}|}\underset{{\text {Metric}}}{\sum }(M((x_i,y_i)) - \mu )^2}$, where $\mu$ is the mean.

### Performance comparison

**Fig. 2:**
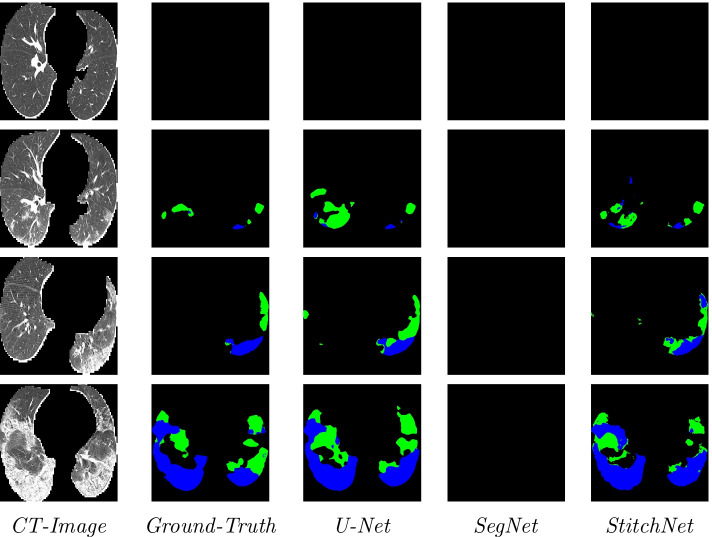
Visual comparison of the segmentation results, where the green and blue labels indicate GGO and Consolidation, respectively

We compared StitchNet to SegNet and to U-Net with a MobileNetV2 encoder. The hyperparameters used to train StitchNet can be found in Additional file 1: Table S1. The results on the test set are shown in Table 1 with some example prediction shown in Fig. 2. The results on the validation and training set can be found in Additional file 1: Tables S2 and S3. Although performance of StitchNet and U-Net are comparable when predicting the ground glass opacity label, StitchNet’s precision is higher whereas U-Net’s recall is higher. This seems to indicate that StitchNet makes more conservative predictions than U-Net. The SegNet fails to predict any lesions, predicting only the background class. This is likely due to the fact that its backbone is based off the outdated VGG16 network [27], whereas StitchNet and U-Net’s backbone uses the more sophisticated MobileNetV2.

**Table 1:** Quantitative results of ground-glass opacity (GGO), consolidation (CON), background, and the overall average on the test dataset

Lesion	Method	IoU	F1	Recall	Precision
GGO	U-Net	0.391 ± 0.280	0.499 ± 0.32	0.608 ± 0.358	0.47 ± 0.326
GGO	SegNet	0.004 ± 0.027	0.007 ± 0.044	0.012 ± 0.087	0.009 ± 0.071
GGO	StitchNet	0.358 ± 0.257	0.471 ± 0.303	0.517 ± 0.331	0.489 ± 0.328
CON	U-Net	0.404 ± 0.331	0.49 ± 0.368	0.616 ± 0.378	0.485 ± 0.38
CON	SegNet	0.021 ± 0.113	0.027 ± 0.137	0.057 ± 0.227	0.021 ± 0.114
CON	StitchNet	0.318 ± 0.315	0.397 ± 0.361	0.539 ± 0.411	0.387 ± 0.369
Background	U-Net	0.983 ± 0.023	0.992 ± 0.012	0.987 ± 0.02	0.996 ± 0.006
Background	SegNet	0.97 ± 0.044	0.984 ± 0.024	0.999 ± 0.009	0.971 ± 0.043
Background	StitchNet	0.985 ± 0.021	0.992 ± 0.011	0.992 ± 0.011	0.993 ± 0.014
Overall	U-Net	0.593	0.66	0.737	0.65
Overall	SegNet	0.332	0.339	0.356	0.334
Overall	StitchNet	0.554	0.62	0.683	0.623

## Discussion

The performance of StitchNet is comparable to that of the fully supervised U-Net model on the ground-glass opacity and consolidation lesion. This indicates that StitchNet is learning from the labelled data, but not the unlabelled data. When trained on only the labelled data, StitchNet predicts styles that are clearly associated with the appropriate lesions, effectively allowing you to see what a CT image would look like if it were entirely filled with the associated lesion. Because of this, StitchNet seems to perform exactly as expected on the labelled data. When trained on only the unlabelled data, StitchNet learns unique and meaningful styles, learning a meaningful clustering of the data. This is exactly what we would expect from training with no labelled data.

Based on these two observations, when trained on both labelled and unlabelled data, StitchNet should learn to predict styles that are associated with lesions, for *both* the labelled data *and* the unlabelled data. StitchNet achieves this on the labelled data, however, on the unlabelled, all the styles are identical, and the segmentations are the exact same for every image. This seems to indicate that the fundamental idea is sound, but that further work needs to be done before StitchNet can outperform the supervised network.

Furthermore, we note that the standard deviation of StitchNet model is consistently lower than that of U-Net model. This is due to the fact that the model is able to reinforce the predictions it makes on the labelled data by training on the unlabelled data, resulting in a model that performs more consistently on unseen data.

## Conclusion

In conclusion, we proposed a novel generative model called the shared variational autoencoder (SVAE), making a theoretical contribution to the field of generative modelling by introducing shared weights between the encoder and the decoder. We used this model to propose StitchNet, a model capable of tackling the challenging task of semi-supervised CT image segmentation. While the theoretical foundation of StitchNet is sound, further work will be needed before it can make full use of unlabelled data.

## Methods

### Shared variational autoencoder

In this section we will introduce the theory, implementation and optimization of the *SVAE*. Suppose we have a dataset, $D = \{x^{(i)}\}_{i = 0}^{N-1}$, of *N* images. Assuming that these images are independent and identically distributed (i.i.d). samples from some ground-truth distribution, *p*(*x*), we wish to approximate that ground truth distribution. This allows us to sample from our approximation, synthesizing new images. The Ladder Variational Autoencoder (LVAE) [26] is a recently proposed model that has been shown to be highly effective at modelling such distributions. Here we will briefly summarize their work and discuss some potential issues. The LVAE assumes that the data is generated in a hierarchical sampling process. Specifically, it assumes that, to generate an image, we first take a sample from a unit Gaussian distributed-latent variable, $z_n$. A function is then applied to that sample, outputting the parameters to a diagonal Gaussian distributed-latent variable, $z_{n-1}$. This will repeat for n levels, with the last output distribution being a distribution for each pixel in the image (their work used a Bernoulli distribution). This allows them to assume independence between the pixels when conditioned on the latent variables. This model is denoted by $p_\theta$.

**Fig. 3:**
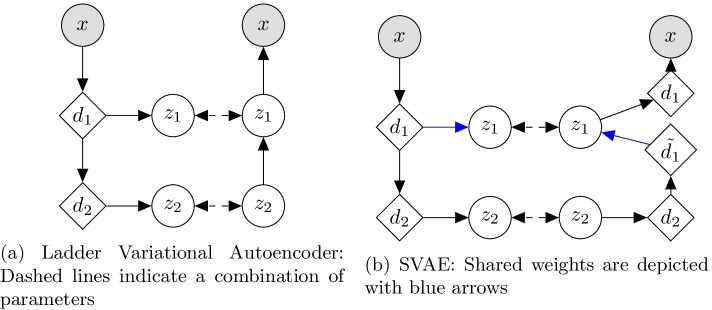
A comparison between the LVAE and our SVAE

The LVAE uses variational inference to learn both the model $p_\theta$ and an approximate posterior to $p_\theta$, $q_\phi$. In previous work, $q_\phi$ will infer the value for $z_1$ from *x* and $z_2$ from $z_1$. The LVAE differs from this in that their $q_\phi$ completes a deterministic down pass, and then each *z* is inferred from the intermediate layers of this down pass. The dependencies between latent variables are recovered by combining the inferred distributions’ parameters for the latent variables with the generative model’s predicted distributions’ parameters. This is depicted in Fig. 3a.

Though the LVAE is quite interesting, it was not designed to work well on large, complex datasets such as of CT images. In this study, we seek to modify the LVAE so that it will work well on such a dataset. We do this by replacing the mappings between latent variables in the LVAE with deep convolutional layers that have been handcrafted to work well on CT images. Now, to find $z_1$ given $z_2$, we apply a deconvolutional layer to $d_2$ to get $d_1$ and then apply many convolutional layers to $d_1$ to get $z_1$. We note that, in both $p_\theta$ and $q_\phi$, we have a mapping between $d_n$ and $z_n$. We hypothesize that this mapping serves the exact same purpose in both, and that having both share weights would increase performance. With this final change, we arrive at the *shared variational autoencoder* (depicted in Fig. 3b)

**Fig. 4:**
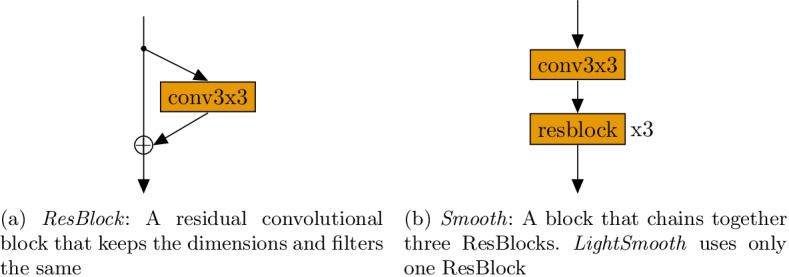
A description of the building blocks of the SVAE

**Table 2:** The dimensionality of the five latent variables

Level	*z*	*d*
0	(352,352,1)	NA
1	(176,176,1)	(176,176,32)
2	(88,88,1)	(88,88,64)
3	(44,44,1)	(44,44,128)
4	(22,22,1)	(22,22,256)
5	(11,11,1)	(11,11,512)

Level 0 denotes the input image *x*

**Fig. 5:**
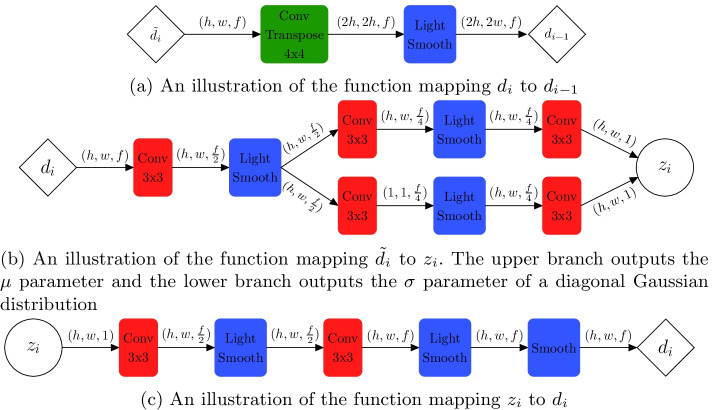
An illustration of the mappings between the SVAE’s variables. We denote the output of each block as $(height, \,width,\,filters)$

Here, we will describe the deep convolutional layers used in the SVAE model. Several building blocks of the model are described in Fig. 4. When the number of filters is greater than 64, linear bottleneck convolutions [28] are used instead of the traditional convolution. Batch Normalization [29] followed by the ReLU [30] activation follows every convolutional layer and is suppressed for clarity. SVAE has five layers of latent variables, opposed to the two depicted in Fig. 3b. The dimensionality of these latent variables and their deterministic expansion is shown in Table 2. We use the intermediate and output layers of *MobileNetV2* [28] with the image, *x*, as input to obtain $d_1,d_2,...d_5$. The mappings between variables are depicted in Fig. 5.

### StitchNet

**Fig. 6:**
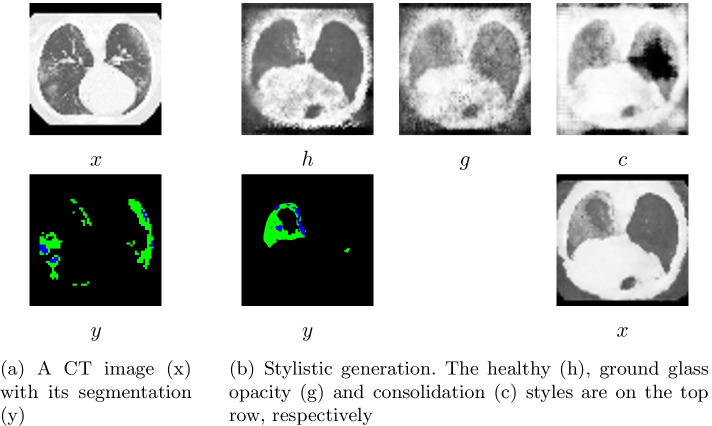
Visualization of the data and StitchNet’s outputs. For segmentations, ground glass opacity is shown in green, consolidation in blue and healthy tissue in black

In this section we will introduce the theory, implementation and optimization of the *StitchNet*. Suppose we have a dataset, $D_{UN} = \{(x^{(i)})\}_{i=0}^{N-1}$, of *N* CT images, where $x^{(i)}$ denotes the $i^{th}$ CT image in the dataset. We will assume that $x^{(i)}$ is a high-dimensional vector with entries ranging from zero to one. Suppose that we have a dataset, $D_{LAB} = \{(x^{(i)},y^{(i)})\}_{i=0}^{M-1}$, of *M* CT images along with their associated segmentation, $y^{(i)}$. We will assume that $y^{(i)}$ is of the same dimension as $x^{(i)}$ and has entries that belong to the set $\{0,1,2,3\}$. Here, if the $n^{th}$ entry of $y^{(i)}$ is equal to zero, then this indicates that the $n^{th}$ entry of $x^{(i)}$ is part of the background of the CT image Furthermore, one, two and three correspond to the healthy tissue, ground-glass opacity and consolidation class, respectively. This is depicted in Fig. 6a.

We wish to obtain a model capable of taking a CT image (*x*) and outputting an accurate segmentation (*y*). In other words, we wish to approximate the ground-truth *p*(*x*|*y*) conditional distribution. Though not typically phrased in these terms, supervised deep-learning techniques do this by introducing the following approximation to this distribution:

2 $$\begin{aligned} p_\theta (y|x) = {\text {CAT}}(y|f_\theta (x)), \end{aligned}$$

where ${\text {CAT}}$ is the Categorical distribution and $f_\theta$ is some complex function. Due to their tremendous success on image data, $f_\theta$ is typically chosen to be a convolutional neural-network.

These supervised techniques then aim to find the parameters $\theta$ that best explains the data we are given. This is done by maximizing the following objective using a numerical approximation algorithm such as gradient descent:

3 $$\begin{aligned} J = \sum _{D_{LAB}} \log p_\theta (y^{(i)}|x^{(i)}). \end{aligned}$$

Phrased in this way, the drawback to these supervised techniques is obvious. They can only use the labelled dataset $D_{LAB}$. To remedy this, instead of approximating *p*(*y*|*x*), we can model joint distribution *p*(*x*, *y*) and derive the conditional distribution *p*(*y*|*x*) from it. This allows us to use both, $D_{LAB}$ and $D_{UN}$ by treating *y* as a latent variable in the latter case.

**Fig. 7:**
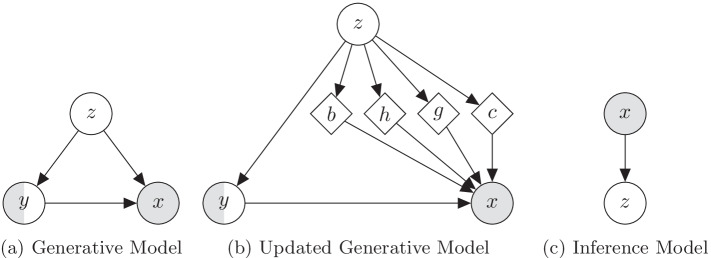
Hierarchical graphical models. Latent, partially observed and observed variables are shown with clear, half-filled and filled, respectively. Arrows and diamond nodes represent functional mappings

To effectively model *p*(*x*, *y*), we will assume that *x* and *y* are dependent on the hierarchy of latent variables from the SVAE, which here we will simply denote as *z*. Now we will model *p*(*x*, *y*, *z*). Furthermore, we will assume that each of the data points, $(x^{(i)},y^{(i)},z^{(i)})$, were generated in the following way:

4 $$\begin{aligned} \begin{array}{r@{}l} z^{(i)} \sim p(z), \,\,\, y^{(i)} \sim p_\theta (y|z^{(i)}), \,\,\, x^{(i)} \sim p_\theta (x|z^{(i)},y^{(i)}), \,\,\, \end{array} \end{aligned}$$

where *p*(*z*) are assumed to follow the distribution from the SVAE and $p_\theta (\cdot )$ is assumed to be some distribution parameterized by $\theta$ (depicted in Fig. 7).

To generate *x* we will first generate four stylistic representations–referred to as *b*, *h*, *g* and *c*–of *x*. These stylistic representations of *x* show you what the image would look like if the entire lung were background, healthy, ground-glass opacity and consolidation, respectively. We then reconstruct *x* by choosing the pixel from the style associated with the label predicted by *y*. Examples of these styles are shown in Fig. 6b.

We will use this, as well as the following definitions, to define $p_\theta$:

5 $$\begin{aligned} \begin{array}{r@{}l} p_\theta (\{b,h,g,c\}|z) &{}= {\text {BETA}}(\{b,h,g,c\}|\alpha _\theta (z),\beta _\theta (z)), \\ \Phi (y,b,h,g,c) &{}= {\left\{ \begin{array}{ll} b &{} {\text {if }}y = 0,\\ h &{} {\text {if }}y = 1,\\ g &{} {\text {if }}y = 2,\\ c &{} {\text {if }}y = 3, \end{array}\right. } \end{array} \end{aligned}$$

where BETA denotes the Beta distribution and $\alpha _\theta (z)$ and $\beta _\theta (z)$ are some complex functions parameterized by $\theta$ which outputs the parameters of the beta distribution.

Finally, we define $p_\theta$ as

6 $$\begin{aligned} \begin{array}{r@{}l} p_\theta (y|z) &{}= {\text {CAT}}(y|\pi _\theta (z)),\\ p_\theta (x|y,b,h,g,c) &{}= {\text {BETA}}(x|\Phi (y,b,h,g,c)), \end{array} \end{aligned}$$

where $\pi _\theta (z)$ is some complex function parameterized by $\theta$. We now have a generative model that is well suited to CT image segmentation (depicted in Fig. 7b, c). What remains is outlining an effective means for finding the values of $\theta$ that best explains our observed data. Concretely, we wish to solve

7 $$\begin{aligned} \begin{array}{r@{}l} \max _{\theta } &{}\sum _{D_{UN}}\log p_\theta (x^{(i)}) + \sum _{D_{LAB}}\log p _\theta (x^{(i)},y^{(i)}) \\ &{}= \sum _{D_{UN}}\log \int \limits _z \sum _y p_\theta (x^{(i)},y,z)dz\\ &{}\quad + \sum _{D_{LAB}}\log \int \limits _z \ p_\theta (x^{(i)},y^{(i)},z)dz. \end{array} \end{aligned}$$

The existence of latent variable, and, by extension, the need to integrate over them, makes this objective completely intractable. We instead optimize a variational lower bound on the log likelihood of $p_\theta$. Concretely, we optimize,

8 $$\begin{aligned} \begin{array}{r@{}l} &{} \log p_\theta (x^{(i)}) \ge E_{q_\phi (z,k|x^{(i)})} \left[ \log \frac{p_\theta (x^{(i)},y^{(i)},z)}{q_\phi (z|x^{(i)})} \right] , \\ &{} \log p_\theta (x^{(i)}) \ge E_{q_\phi (z|x^{(i)})} \left[ log \frac{\sum _y p_\theta (x^{(i)},y,z)}{q_\phi (z|x^{(i)})} \right] . \end{array} \end{aligned}$$

Though $q_\phi$ can be any function of the latent variables, this lower bound is exactly equal to the true log likelihood when $q_\phi$ is equal to $p_\theta 's$ posterior, $p_\theta (z|x,y)$. Therefore, $q_\phi$ has the interpretation of being an approximation to the posterior. When we implement the $q_\phi$, we will keep this fact in mind.

We can further increase tractability by approximating the calculation of the expectation over $q_\phi$. We do this by taking a Monte-Carlo sample from $q_\phi$ and evaluating the expectation with just this sample. This approximation can be made more precise by taking multiple samples and averaging the expectation, but, for our work, we used only one. With this, we arrive at our final objective, which can be optimized via any gradient descent algorithm.

9 $$\begin{aligned} \begin{array}{r@{}l} &{}E_{q_\phi (z|x^{(i)})} \left[ log\frac{p_\theta (x^{(i)},y^{(i)},z)}{q_\phi (z|x^{(i)})} \right] \approx q_\phi (z^{(i)}|x^{(i)})\left[ log\frac{p_\theta (x^{(i)},y^{(i)},z^{(i)})}{q_\phi (z|x^{(i)})} \right] \equiv J_{LAB},\\ &{}E_{q_\phi (z|x^{(i)})} \left[ log \frac{\sum _y p_\theta (x^{(i)},y,z)}{q_\phi (z|x^{(i)})} \right] \approx q_\phi (z^{(i)}|x^{(i)})\left[ log \frac{\sum _y p_\theta (x^{(i)},y,z^{(i)})}{q_\phi (z|x^{(i)})} \right] \equiv J_{UN},\\ &{}{\text {where}} \,\, z^{(i)} \sim q_\phi (z|x^{(i)}). \end{array} \end{aligned}$$

## Supplementary Information

**Additional file 1**: **Table S1**. The chosen hyperparameters used to train StitchNet. **Table S2**. Quantitative results of ground-glass opacity (GGO), consolidation (CON), background, and the overall average on the validation dataset. **Table S3**. Quantitative results of ground-glass opacity (GGO), consolidation (CON), Background, and the overall average on the training dataset.

## Data Availability

The CC-CCII dataset used for our analysis can be found at http://ncov-ai.big.ac.cn/download?lang=en. All code necessary for the implementation of StitchNet and the replication of our results can be found at https://github.com/JudahZammit/stitchnet.

## Abbreviations

COVID-19: Coronavirus disease 2019
CT: Computed tomography
SVAE: Shared variational autoencoder
LVAE: Ladder variational autoencoder
VAE: Variational autoencoder
CC-CCII: China Consortium of Chest CT Image Investigation
CP: Common pneumonia
NCP: Novel-coronavirus pneumonia
