# Supplementary material for: Semi-supervised COVID-19 CT image segmentation using deep generative models
Source: BMC Bioinformatics. 2022 Aug 17;23(Suppl 7):343. doi: 10.1186/s12859-022-04878-6 (PMC9381397; doi:10.1186/s12859-022-04878-6)
Supplement: Supplementary file 1 — Additional file 1: Table S1. The chosen hyperparameters used to train StitchNet. Table S2. Quantitative results of ground-glass opacity (GGO), consolidation (CON), background, and the overall average on the validation dataset. Table S3. Quantitative results of ground-glass opacity (GGO), consolidation (CON), Background, and the overall average on the training dataset. [file 12859_2022_4878_MOESM1_ESM.pdf]

Supplementary

Table S1: The chosen hyperparameters used to train StitchNet

**Hyper-Parameter**	**Value**
Optimizer	ADAM [31]
Learning Rate	1e-4
Gradient Clipping Norm	1
Gradient Clipping Value	0.5
Epochs	80
Batch Size	3
Healthy/NCP/Unlabelled Ratio	1:1:1
Image Dimension	352 X 352
Data Augmentation	{Random Crop, Random Flip}

Table S2: Quantitative results of Ground-Glass Opacity (GGO), Consolidation (CON), Background, and the overall average on the validation dataset

Methods	Lesion		IoU	F1	Recall	Prec
U-Net	GGO	Mean	0.304	0.405	0.544	0.365
		STD	± 0.259	± 0.308	± 0.373	± 0.302
SegNet		Mean	0.003	0.005	0.009	0.007
		STD	± 0.027	± 0.042	± 0.066	± 0.068
**StitchNet**		Mean	0.309	0.418	0.487	0.427
		STD	± 0.243	± 0.296	± 0.334	± 0.325
U-Net	CON	Mean	0.352	0.426	0.533	0.464
		STD	± 0.343	± 0.376	± 0.402	± 0.397
SegNet		Mean	0.018	0.022	0.026	0.018
		STD	± 0.105	± 0.128	± 0.154	± 0.109
**StitchNet**		Mean	0.278	0.348	0.455	0.349
		STD	± 0.313	± 0.362	± 0.417	± 0.381
U-Net	Background	Mean	0.984	0.992	0.987	0.996
		STD	± 0.023	± 0.012	± 0.02	± 0.006
SegNet		Mean	0.974	0.987	0.999	0.975
		STD	± 0.036	± 0.019	± 0.004	± 0.036
**StitchNet**		Mean	0.987	0.993	0.991	0.996
		STD	± 0.017	± 0.009	± 0.012	± 0.008
U-Net	Overall	Mean	0.547	0.608	0.688	0.608
		Error	N/A	N/A	N/A	N/A
SegNet		Mean	0.332	0.338	0.345	0.333
		Error	N/A	N/A	N/A	N/A
**StitchNet**		Mean	0.525	0.586	0.644	0.591
		Error	N/A	N/A	N/A	N/A

Table S3: Quantitative results of Ground-Glass Opacity (GGO), Consolidation (CON), Background, and the overall average on the training dataset

Methods	Lesion		IoU	F1	Recall	Prec
U-Net	GGO	Mean	0.472	0.573	0.696	0.526
		STD	± 0.303	± 0.334	± 0.368	± 0.329
SegNet		Mean	0.004	0.007	0.006	0.008
		STD	± 0.033	± 0.051	± 0.045	± 0.065
**StitchNet**		Mean	0.39	0.508	0.571	0.517
		STD	± 0.256	± 0.295	± 0.327	± 0.312
U-Net	CON	Mean	0.561	0.647	0.69	0.652
		STD	± 0.328	± 0.349	± 0.349	± 0.362
SegNet		Mean	0.01	0.013	0.023	0.011
		STD	± 0.077	± 0.096	± 0.14	± 0.082
**StitchNet**		Mean	0.395	0.485	0.564	0.482
		STD	± 0.318	± 0.36	± 0.386	± 0.365
U-Net	Background	Mean	0.991	0.995	0.992	0.998
		STD	± 0.011	± 0.006	± 0.01	± 0.002
SegNet		Mean	0.972	0.986	0.999	0.973
		STD	± 0.037	± 0.02	± 0.008	± 0.036
**StitchNet**		Mean	0.988	0.994	0.992	0.996
		STD	± 0.016	± 0.008	± 0.011	± 0.008
U-Net	Overall	Mean	0.675	0.738	0.793	0.725
		Error	N/A	N/A	N/A	N/A
SegNet		Mean	0.329	0.335	0.343	0.331
		Error	N/A	N/A	N/A	N/A
**StitchNet**		Mean	0.591	0.662	0.709	0.665
		Error	N/A	N/A	N/A	N/A
